# HDAC3 knockdown inhibits ferroptosis via upregulating Nrf2 to alleviate renal interstitial fibrosis in lupus nephritis

**DOI:** 10.1136/lupus-2025-001666

**Published:** 2025-10-22

**Authors:** Tianli Shi, Zhen Luo, Wenjuan Lei

**Affiliations:** 1 Department of Nephropathy and Rheumatology, Haikou People’s Hospital, Haikou, Hainan, China

**Keywords:** Lupus Nephritis, Antibodies, Inflammation

## Abstract

**Background:**

Lupus nephritis (LN) is the most serious complication of SLE. Interstitial fibrosis is the dominant pathological change of renal injury in LN. Enhanced histone deacetylase 3 (HDAC3) positively correlates with renal interstitial fibrosis (RIF). Our study objective was to explore the accurate role and mechanism of HDAC3 in the RIF of LN.

**Methods:**

To knock down HDAC3, Murphy Roths large (MRL)/wt and MRL/MpJ-Faslpr/J (MRL/lpr mice were injected with lentiviral short hairpin RNAs. Human renal proximal tubular epithelial cells (HK-2 cells) were treated with serum from patients with LN to establish an LN cell model. Renal histopathological change was assessed by H&E, Masson and Sirius red staining. Cell viability was determined using Cell Counting Kit-8 (CCK-8) kits. Inflammation cytokine determination was conducted employing ELISA assays. Protein expression was detected by western blot, and immunohistochemical and immunofluorescence staining. Gene densities were analysed by quantitative real-time PCR assays. Co-immunoprecipitation analysis validated the interactions of nuclear factor erythroid 2-related factor 2 (Nrf2) and Kelch-like ECH-associated protein 1 (Keap1).

**Results:**

HDAC3 levels were increased in the serum and kidney tissues of patients with SLE and LN, and the LN group posted the highest level. HDAC3 knockdown ameliorated RIF, oxidative stress, inflammation and ferroptosis in kidney tissues of MRL/lpr mice. Moreover, HDAC3 inhibition repressed the inflammatory and oxidative reactions, fibrosis and ferroptosis in LN-serum-induced HK-2 cells. Further, HDAC3 knockdown could inhibit the Keap1-Nrf2 interaction to trigger Nrf2 activation.

**Conclusion:**

HDAC3 inhibition relieved RIF, oxidative stress, inflammation and ferroptosis by upregulating Nrf2 in the mice and cell models of LN.

**WHAT IS ALREADY KNOWN ON THIS TOPIC**

- Renal interstitial fibrosis (RIF) can serve as a marker of the severity of renal injury in lupus nephritis (LN), and histone deacetylase 3 (HDAC3) expression is positively correlated with the progression of fibrosis.

**WHAT THIS STUDY ADDS**

- Inhibition of HDAC3 alleviated RIF, oxidative stress, inflammatory injury and ferroptosis by enhancing nuclear factor erythroid 2-related factor 2 (Nrf2) in both murine and cellular models of LN.

**HOW THIS STUDY MIGHT AFFECT RESEARCH, PRACTICE OR POLICY**

- The identified mechanism of HDAC3 and its interaction with Nrf2 have enriched the understanding of the pathogenesis of LN and can be used for early diagnosis and intervention.

## Introduction

 SLE is a critical autoimmune disease affecting multiple organ systems, leading to a range of clinical symptoms.^1^ Approximately 50% of patients with SLE experience kidney tissue injury, with lupus nephritis (LN) being a common and serious manifestation of SLE, closely associated with end-stage renal disease (ESRD).^2^ Clinically, patients with LN typically present with proteinuria, haematuria and, in severe cases, rapidly progressive glomerulonephritis.^3^ Sustained or resurgence LN can progress to renal inadequacy and failure, which significantly impacts the health and life quality of patients with LN.^4^ Conventional therapies for LN consist of two stages: remission induction and maintenance treatment, aimed at alleviating renal injury and restoring kidney function.^5^ Current pharmacological options for LN treatment include glucocorticoids, hydroxychloroquine and immunosuppressive agents such as cyclophosphamide; however, their efficacy and stability remain limited.^6^,^8^ Moreover, the corresponding pathology mechanisms remain elusive and insufficient to identify the accurate targets for LN controlling. Given the severity of LN, it is urgent to explore the fundamental molecular mechanisms and search for potentially relevant targets for clinical utilisation.

Renal interstitial fibrosis (RIF) is contended as the primary pathological change and injury marker in the kidney tissues of patients with LN.^9^ Furthermore, RIF represents the final manifestation of ESRD and can significantly influence the progression of LN to ESRD.^10 11^ Therefore, a deeper understanding of the mechanisms underlying RIF can be instrumental in managing the LN process. The typical histopathological features of RIF include the deposition of extracellular matrix components, such as collagen I (COL-I) and collagen III (COL-III), a reduction in tubular cells, an increase in fibroblast populations and a decrease in capillary density around the tubules.^12^ Among the potential causes of RIF, the epithelial-mesenchymal transition of renal tubular epithelial cells is considered the dominant factor.^13^ Transforming growth factor-β (TGF-β), Snail family zinc finger 1 and Zinc finger protein SNAI2 (SLUG) serve as key regulators in promoting epithelial-mesenchymal transition (EMT) progression.^14 15^ In addition, the non-revolving inflammation, characterised by the excessive enrichment of inflammatory cells and their released cytokines, is a vital promotive factor in RIF progression.^16^ Oxidative stress in renal tubular epithelial cells is another significant contributor to the development of RIF.^17^ Although the upstream mechanisms underlying renal collagen fibre deposition, inflammation and oxidative stress in RIF remain unclear, further exploration of these pathways will be beneficial for controlling RIF and LN progression.

Histone deacetylase 3 (HDAC3) is a crucial member of the HDAC family, closely linked with the development, function and health of the kidney.^18^ As a modulatory protein, HDAC3 participates in regulating the progression of renal diseases, including kidney cancer, renal fibrosis and diabetic nephropathy, with its inhibitors demonstrating a renal protective role.^18 19^ In a chronic kidney disease mouse model, the expression level of HDAC3 is significantly upregulated, and its inhibition can alleviate renal fibrosis.^20^ Further, the depletion of HDAC3 markedly restrains the hyperuricaemia-induced RIF in rat models.^21^ Additionally, HDAC3 inhibition has been shown to reduce abnormal inflammation associated with renal fibrosis; however, its modulatory effects on oxidative stress remain unreported.^20 22^ The precise role and mechanisms of HDAC3 in LN and the internal regulation of RIF are unknown and require further exploration.

Therefore, we designed to clarify the role and mechanism of HDAC3 in RIF using both animal and cellular LN models. We found that HDAC3 expressions were dramatically elevated in serum and kidney tissues of patients with LN. Inhibition of HDAC3 was found to alleviate RIF, oxidative and inflammatory injury, and ferroptosis by promoting the activity of nuclear factor erythroid 2-related factor 2 (Nrf2) in both mouse and cell models of LN. The mechanisms we have elucidated regarding HDAC3 inhibition may have implications for clinical treatment of LN, and the interaction between HDAC3 and Nrf2 could serve as a potential target for early diagnosis and intervention in LN.

## Material and methods

### Ethics statement and clinical samples

The patients with LN (n=22) were classified as the LN group, comprising 10 men and 12 women, with a mean age of 29.82±3.10 years. In addition, the SLE group (n=22) included 9 men and 13 women, with a mean age of 33.10±4.87 years. The control group (n=22) consisted of 22 healthy participants, comprising 13 men and 9 women, with a mean age of 34.55±7.42 years. The clinical and histological definitions of LN included: proliferative changes in glomerular cells; fibrinoid necrosis and nuclear dissolution; cellular crescents; wire loop phenomenon and hyaline thrombus; neutrophil infiltration in glomeruli; mononuclear cell infiltration in renal interstitium.^23^ The levels of proteinuria and the main corresponding clinical manifestations of LN cases are described in the online supplemental table S3. The patients’ inclusion and exclusion criteria were compatible with the previous standards.^24^ The inclusion criteria included: (1) >18 years old; (2) With no psychiatric illness history; (3) With no uric acid lowering, renal-protecting, diuretics and other medications; (4) Fulfilling SLE diagnostic criteria of the American College of Rheumatology instruction and renal injury criteria: urinary protein >0.5 g/day or tubular urine, erythrocyte cast, mixed tubular elements or haemoglobin granules (specific for patients with LN). The exclusion criteria included: (1) With cardiovascular disorders, autoimmune conditions, malignant tumours, acute or chronic infectious diseases; (2) With alternative primary or secondary renal pathologies; (3) Pregnant or breastfeeding; (4) Received immunosuppressor or high-dose corticosteroid therapy. Further, the biopsied kidney tissues and blood samples were collected from patients with LN, patients with SLE and controls for analysis of pathological parameters and determination of signalling molecule.^23^ All patients admitted to our project understood the research situation and signed informed consent.

### Serum analysis

Elbow venous blood was obtained from the participants (LN, SLE and control groups) and then centrifuged at 1500 g for 15 min to extract the serum samples at 4°C.^24^ Serum samples were used to detect the concentrations of HDAC3 (#P-4040–96, Epigentek, Farmingdale, New York, USA), Nrf2 (#ab277397, Abcam, Shanghai, China) and glutathione peroxidase 4 (GPX4) (#ab304936, Abcam) using the corresponding ELISA kits, based on the respective kit’s protocol. The relevant absorbance was read on a microplate reader (Synergy HT, BioTek, Winooski, Vermont, USA).

### Animals experiment

Murphy Roths large (MRL)/wt and MRL/MpJ-Faslpr/J (MRL/lpr) female mice (20±2 g of body weight (BW)) were obtained from Viewsolid Biotech (Beijing, China) and maintained in the animal room (12 hours light/darkness cycle, 23°C, 50% humidity), with water and feed ad libitum. To knock down HDAC3 in MRL/lpr female mice, lentiviral short hairpin RNA targeting HDAC3 (lenti-shHDAC3, 10^9^ TU/ml) and their negative control (lenti-shNC) were acquired from Hanbio Biotech (Shanghai, China) and these vectors were tail-intravenous injected into mice (2.5×10^8^ relative infectious unit (R.I.U)) once a week for 2 weeks, at 10 weeks, following our preliminary experiment data and relevant studies.^25 26^ At 24 hours after the last administration, the kidney tissues of mice were collected for histological observation, protein and gene expression detection, oxidative marker levels evaluation and inflammatory cytokine levels assessment. Furthermore, to investigate the impact of HDAC3 silencing on mouse survival duration, the survival period was recorded until 45 weeks to obtain the Kaplan-Meier survival curve. All the animal experiment protocols were performed in accordance with the Animal Research: Reporting of in vivo Experiments (ARRIVE) guidelines.

### Renal histopathological analysis

Collected renal tissues were fixed with paraformaldehyde (4%) for 48 hours, dehydrated and embedded in paraffin, following the operational method.^27^ Renal sections received H&E staining (4 µm thickness), Masson’s trichrome staining (4 µm thickness) and Sirius red staining (6 µm thickness), respectively. All the staining procedures followed the staining kits’ guidelines. In brief, for H&E staining, sections were incubated with haematoxylin (1 min) and eosin resolution (30 s), using an H&E stain kit (#G1120, Solarbio Biotech, Beijing, China). For Masson’s trichrome staining, kidney tissue sections were incubated with Masson complex (5 min), Masson blue solution (2 min), Ponceau S red magenta solution (5 min), phosphomolybdic acid solution (5 min) and aniline blue solution (3 min) from the modified Masson’s trichrome stain kit (#G1346, Solarbio). The image’s blue pixel was photographed to measure renal fibrosis. Moreover, the sections were incubated with the staining drops for 1 hour and Mayer’s haematoxylin solution for 10 min, employing the modified Sirius red stain kit (No Picric Acid, #G1472, Solarbio). Five differential images were selected to observe the pathological morphology and the collagen fibre deposition in the renal interstitium under the Leica DM500 light and DM6 B polarising microscope (Leica, Shanghai, China). The Image J (National Institutes of Health (NIH), Bethesda, Maryland, USA) and Image-Pro Plus software (Media Cybernetics, Rockville, Maryland, USA) were employed to analyse the collagen fibre content in renal sections. Interstitial fibrosis scores were evaluated blindly by the same pathologist, as previously instructed.^28 29^

### Immunohistochemical staining

Renal tissue paraffin sections were subjected to dewaxing and dehydration, followed by incubation with the target antibodies, including antitype I collagen (COL-I, #ab308455, 1:1000, Abcam), antifibronectin (#ab2413, 1:1000, Abcam), anticaspase 3 (#ab32351, 1:250, Abcam) and anti-IgG (#ab172730, 1: 250, Abcam) antibodies for 12 hours at 4℃. After washing with phosphate buffered saline (PBS) for 15 min, the sections received the relevant secondary antibody incubation for 1 hour at 25℃. Subsequently, the corresponding protein expression was monitored using diaminobenzidine under a Leica DM2500 microscope (Leica). Image J software was used to statistically analyse the high-power fields of positively stained cells.

### Cell culture and treatment

Human renal proximal tubular epithelial cells (HK-2 cells) were acquired from the American Type Culture Collection (Manassas, Virginia, USA) and cultured in Dulbecco’s Modified Eagle Medium (Gibco, Indianapolis, Indiana, USA) supplementing fetal bovine serum (FBS, 10%) and 1% Penicillin/Streptomycin (P/S), at 37°C and with 5% CO_2_, in a standard humidified incubator. To conduct the LN in vitro model, HK-2 cells were stimulated with the serum of patients with LN (replacing the 25% of FBS, LN-Serum group), and the control group was not changed, using the normal FBS.

### Cell transfection

To silence HDAC3 expression in HK-2 cells, the targeting lenti-shHDAC3 and lenti-shNC obtained from Hanbio Biotech were transduced into HK-2 cells employing the lentiviral packaging plasmid mix (#HB-LLF-1000, Hanbio Biotech) for 24 hours, guided by the kit’s instructions. After that, the infection efficacy was validated by detecting HDAC3 mRNA expression in HK-2 cells.

### Cell viability (cell proliferation) assays

The cell viability of HK-2 cells was assessed using a cell counting kit (#40 203ES60, Cell Counting Kit-8 (CCK-8), Yeasen Biotech, Shanghai, China). Briefly, cells (4×10^3^ cells/well) were cultivated in 96-well plates for 24 hours. After the corresponding treatment, the CCK-8 solution (10 µL) was supplemented into each well and incubated for three more hours. Then, the absorbance value was read at 450 nm on a microplate reader (BioTek).

### ELISA

As previously described, ELISA assays were used to evaluate the HDAC3, Nrf2 and GPX4 levels in serum of controls and patients with SLE and LN. Moreover, the proinflammatory cytokine levels in HK-2 cell culture supernatant, including interleukin-1β (IL-1β, #437004, BioLegend, San Diego, California, USA) and IL-6 (#430504, BioLegend), and the profibrotic cytokine TGF-β (#432907, TGF-β, BioLegend) were also measured using ELISA kits, following the kits’ guidelines. Finally, the relevant absorbance was read on a BioTek microplate reader.

### Measurement of oxidative stress and ferroptosis markers

The levels of malondialdehyde (MDA, #S0131M, Beyotime Biotech, Shanghai, China) and glutathione (GSH, #S0053, Beyotime), as well as the activities of the enzymes catalase (CAT, #S0051, Beyotime) and superoxide dismutase (SOD, #S0101M, Beyotime), were determined using commercial kits according to the protocols provided by the manufacturers. Reactive oxygen species (ROS, #S0035S, Beyotime) levels in the HK-2 cell culture supernatant were also assessed using the relevant kit. Moreover, the ferroptosis marker levels of 4-hydroxynonenal (4-HNE, #ab238538, Abcam) and iron ions (#ab83366, Abcam) in cellular supernatant were detected by corresponding kits.

### Immunofluorescence staining

HK-2 cells were fixed with 4% paraformaldehyde (30 min) and permeabilised with 0.25% Triton X-100 solution (5 min). After washing with PBS, cells were stained with anti-Nrf2 (#ab62352, 1:200, Abcam) and antifibronectin (#ab2413, 1:1000, Abcam) antibodies for 12 hours at 4℃. After that, cells were washed with PBS, incubated with the corresponding secondary antibodies for 1 hour at room temperature and stained with 4’,6-diamidino-2-phenylindole (DAPI) solution (#C0065, Solarbio) for 10 min. Then, the cellular images were visualised under a confocal fluorescence microscope (STELLARIS 8, Leica).

### Quantitative real-time PCR

Total RNA extraction of human kidney tissues, mice kidney tissues and HK-2 cells was conducted employing the total RNA extraction kit (#HXR8076, Huaxingbo Biotech, Beijing, China). Then, the RNA transcription to cDNA and relative mRNA levels were carried out with a FastKing One-Step real-time quantitative-PCR (RT-QPCR) Kit (SYBR Green (SYBR), #FP313, Tiangen Biotech, Beijing, China) on the QuantStudio 12K Flex RT-PCR system (Applied Biosystem, Shanghai, China). glyceraldehyde-3-phosphate dehydrogenase (GAPDH) was chosen as the normalisation control. 2^-∆∆Ct^ was used to calculate the relative change of the target genes. The primer sequences of the present study were exhibited in the online supplemental table S5.

### Western blot analysis

Total protein extraction kits (#P0033, Beyotime) were used to extract the protein from kidney tissues and HK-2 cells. Then, the protein concentration was assessed using an enhanced bicinchoninic acid (BCA) protein assay kit (#P0010S, Beyotime). Protein extracts (15 µg) were subjected to 15% sodium dodecyl sulfate-polyacrylamide gel electrophoresis and subsequently transferred to polyvinylidene difluoride (PVDF) membranes (Millipore, Billerica, Massachusetts, USA) at a temperature of 4°C. These PVDF membranes were immersed in a 5% non-fat milk solution for 1 hour at 25°C. Afterwards, the membranes were incubated with primary antibodies for 12 hours at 4°C. Following this, secondary antibodies conjugated with horseradish peroxidase were used on the membranes for an additional hour at 25°C. The chemiluminescent signals from the protein bands were detected using a BeyoECL Plus system (#P0018S, Beyotime) in conjunction with the ImageQuant LAS system (GE Healthcare, Sunnyvale, California, USA). Anti-HDAC3 (1:1000, #ab32369), anti-GPX4 (1:1000, #ab125066), anti-solute carrier family 7, (cationic amino acid transporter, y+ system) member 11 (SLC7A11) (1:1000, #ab300667), anti-Nrf2 (1:1000, #ab6235), anti-heme oxygenase 1 (HO-1) (1:1000, #ab305290) and anti-GAPDH (1:5000, #ab8245) primary antibodies were acquired from Abcam.

### Co-immunoprecipitation assay

HK-2 cellular lysates were incubated with Protein A/G magnetic beads (Catalogue #HY-K0202, MedChemExpress, Shanghai, China) at 4°C for 12 hours. Subsequently, the samples were incubated overnight at 4°C with anti-Nrf2 antibody (Catalogue #ab6235, Abcam) and IgG antibodies (Catalogue #ab171870, Abcam). Following incubation, the magnetic beads were washed and boiled, and the supernatants were analysed for the expression of Kelch-like ECH-associated protein 1 (Keap1) (Catalogue #ab119403, Abcam) using western blot analysis.

### Statistical analysis

Data analysis was conducted using SPSS V.20.0 (SPSS, Chicago, Illinois, USA). To compare two groups, the Student’s t-test was applied, whereas one-way analysis of variance, followed by Tukey’s test, was employed for the analysis of multiple groups. The resulting mean values were presented together with their respective SDs. Each experiment was independently repeated a minimum of three times. A value of p<0.05 was deemed to indicate statistical significance for the experimental results.

## Results

### Expression pattern and clinical significance of HDAC3 in patients with SLE combined with LN

**Figure 1:**
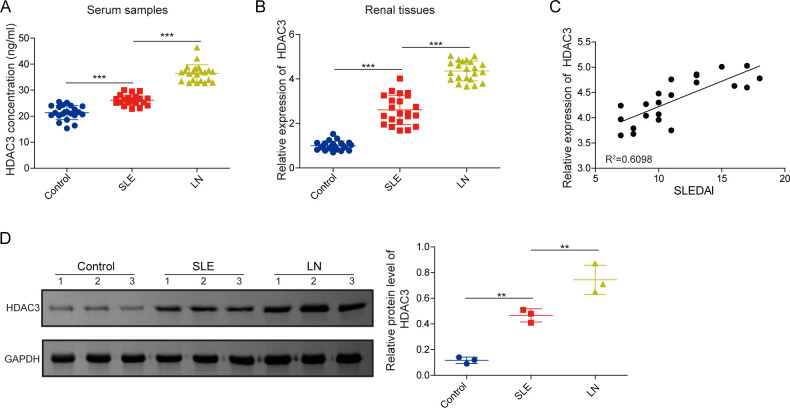
Expression pattern and clinical significance of HDAC3 in patients with SLE combined with LN. (**A**) Serum HDAC3 levels of patients with SLE, patients with LN and control patients were evaluated using ELISA assays. (**B**) Renal HDAC3 gene expressions of patients with SLE, patients with LN and control patients were determined by qRT-PCR analysis. (**C**) Correlation analysis between renal HDAC3 expressions and SLEDAI Scores in the LN group. (**D**) Renal HDAC3 protein expressions of patients with SLE, patients with LN and control patients were detected employing western blot analysis. The results obtained are displayed as mean values±SD. The levels of significance were defined as follows: **p<0.01 and ***p<0.001. ELISA, enzyme-linked immunosorbent assay; GAPDH, glyceraldehyde-3-phosphate dehydrogenase; HDAC3, histone deacetylase 3; LN, lupus nephritis; qRT-PCR, quantitative real-time PCR; SLEDAI, Systemic Lupus Erythematosus Disease Activity Index.

To explore the modulatory function and mechanism of HDAC3 in LN, we analysed the expression pattern of HDAC3 in healthy, SLE and SLE combined with LN patient groups. As described in online supplemental table S1, no substantial variations were noted concerning sex, disease progression, body mass index, dietary habits, smoking history, education, alcohol consumption and other general characteristics across the three groups, aside from the statistically significant age difference (p<0.05). Moreover, we compared the clinical symptoms and pathological parameters between patients with SLE and LN, which indicated that rash, leucopenia, anaemia, serum creatinine, erythrocytopenia, reduced complement C4, ANA, ANA spot and different nucleolus types, showed no significant differences (p>0.05). While significant differences were observed in terms of fever, arthralgia, serum uric acid levels, thrombocytopenia, decreased complement C3, and ANA homogeneous and peripheral types, along with anti-double-stranded DNA (anti-dsDNA) antibodies, anti-Smith antibodies (anti-SMS) and anti-neutrophil cytoplasmic antibodies (ANCA) (p<0.05) (online supplemental table S2). Subsequently, compared with the control group, the HDAC3 levels in the serum of the SLE and LN groups were significantly upregulated, and the HDAC3 levels of the LN group were higher than in the SLE group (figure 1A). In line with that, the HDAC3 mRNA expression levels in renal tissues of patients with SLE and LN were higher than in the control group, and the HDAC3 was most expressed in the LN group (figure 1B). Next, based on the correlation analysis, the renal HDAC3 mRNA levels in the LN group were positively correlated with the Systemic Lupus Erythematosus Disease Activity Index (SLEDAI) Scores, a disease activity index (figure 1C). In addition, the HDAC3 protein densities in the kidney tissues of the SLE and LN groups were significantly higher than the control group, and the LN group posed the highest expression level (figure 1D). Collectively, these fundamental data indicated that HDAC3 was highly expressed in patients with SLE, especially in patients with LN, showing a potential regulatory function in the disease progression of LN.

### HDAC3 knockdown alleviated RIF in MRL/lpr mice

**Figure 2:**
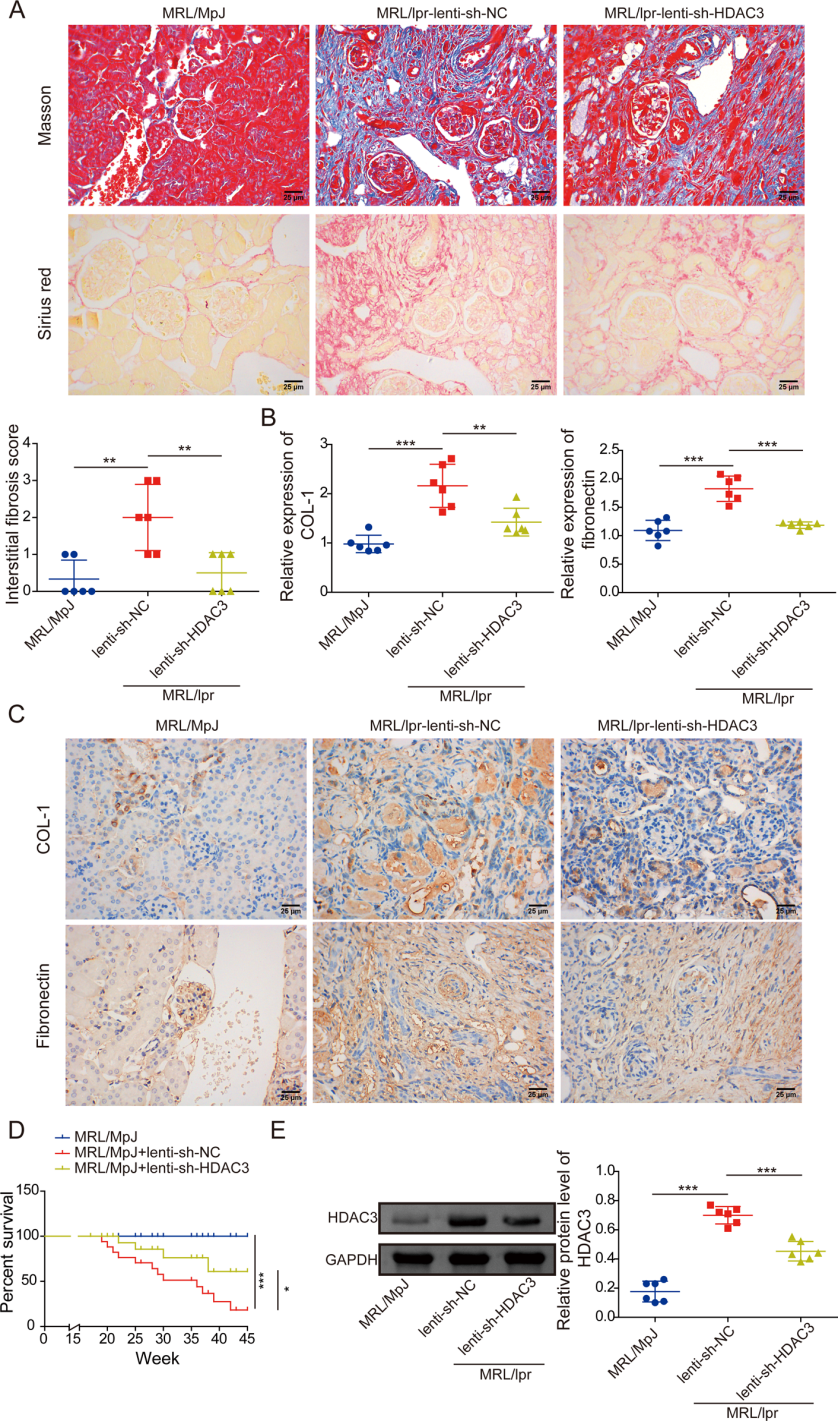
HDAC3 knockdown alleviated renal interstitial fibrosis in MRL/lpr mice. MRL/lpr mice were tail-intravenous injected with lenti-shHDAC3 or lenti-shNC. (**A**) Renal collagen fibres deposition was monitored by Masson and Sirius staining. (**B**) COL-I and fibronectin gene expressions were determined by qRT-PCR analysis. (**C**) COL-I and fibronectin protein expressions were observed by IHC analysis. (**D**) Survival curve of mice with no, lenti-shHDAC3 and lenti-shNC treatment. (**E**) Renal HDAC3 protein expressions were detected employing western blot analysis. The results obtained are displayed as mean values±SD. The levels of significance were defined as follows: *p<0.05, **p<0.01and ***p<0.001. COL-I, collagen I; GAPDH, glyceraldehyde-3-phosphate dehydrogenase; HDAC3, histone deacetylase 3; IHC, immunohistochemical; lenti-shHDAC3, lentiviral short hairpin RNA targeting HDAC3; lenti-shNC, lentiviral short hairpin RNA targeting negative control; MRL, Murphy Roths large; MRL/lpr, MRL/MpJ-Faslpr/J; qRT-PCR, quantitative real-time PCR.

To clarify the role and mechanism of HDAC3 in RIF of LN, MRL/lpr mice were administered tail-intravenous injections with lenti-shHDAC3 or lenti-shNC to knock down HDAC3. As shown in figure 2A and online supplemental table S4, HDAC3 knockdown significantly downregulated the RIF and tubular injury, and it was displayed in the Interstitial Fibrosis Score analysis, from Masson staining analysis, and HDAC3 inhibition restored the body weight, Organ Index, blood counting and renal function in MRL/lpr mice (after 10 weeks’ administration), compared with the lenti-shNC group. Furthermore, Sirius red staining analysis showed that HDAC3 inhibition markedly reduced the collagen fibre deposition of the renal interstitium, in contrast to MRL/lpr mice with lenti-shNC infection (figure 2A). Consistently, knockdown of HDAC3 significantly decreased the RIF markers of COL-I and fibronectin mRNA and protein levels in the kidney tissues (figure 2B,C). Moreover, based on survival time analysis, MRL/lpr mice in the lenti-shHDAC3 treatment group had a longer survival time than the vehicle mice, from the twenty-second week to the forty-fifth week (figure 2D). Subsequently, compared with MRL/MpJ mice, HDAC3 protein expression was significantly upregulated in the kidney tissues of MRL/lpr mice. In contrast, the lenti-shHDAC3 infection in MRL/lpr mice markedly downregulated that expression, suggesting an ideal infection efficacy (figure 2E). In summary, this section's results demonstrated that HDAC3 knockdown could ameliorate RIF and prolong survival time of MRL/lpr mice.

### HDAC3 knockdown mitigated proinflammatory and oxidative injury in MRL/lpr mice

**Figure 3:**
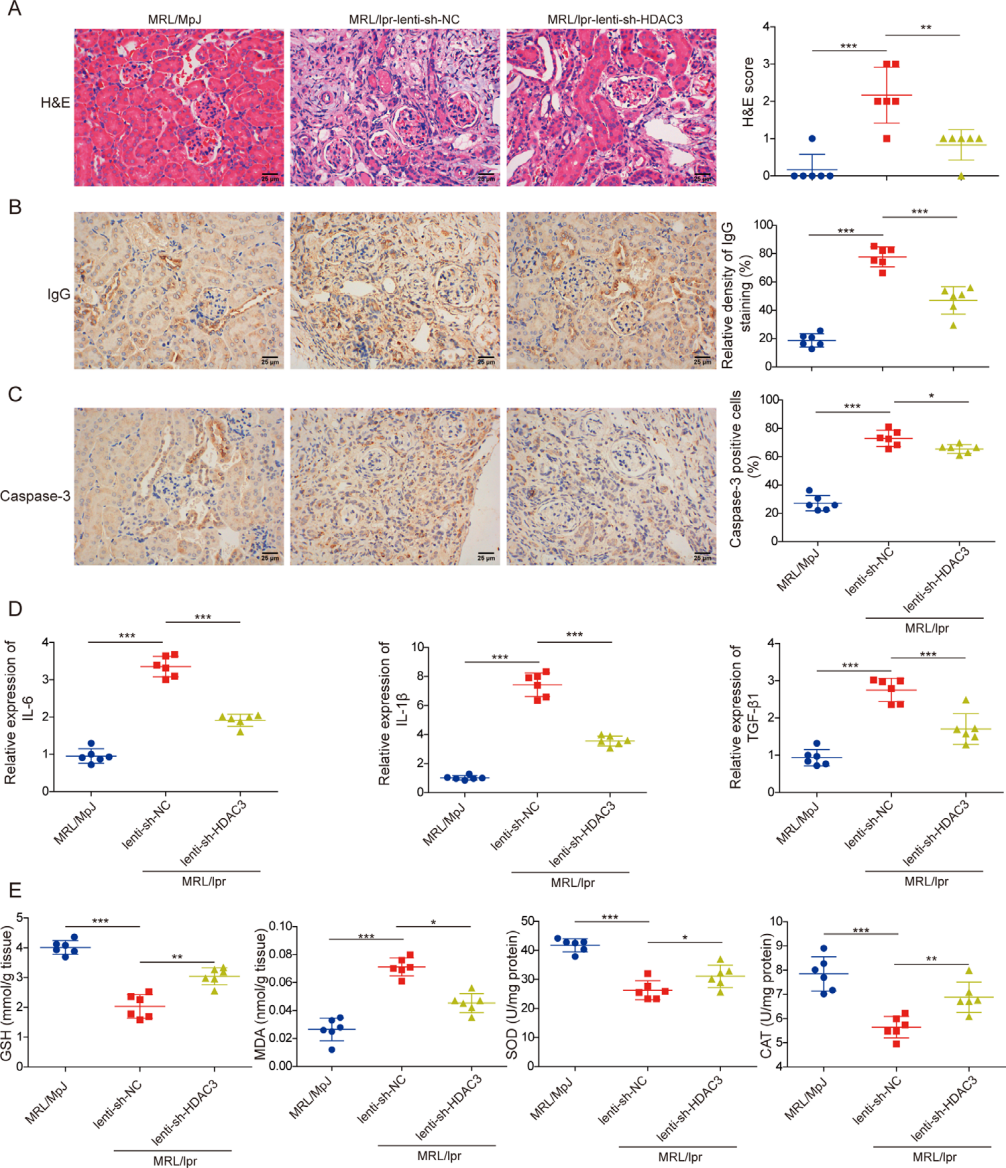
HDAC3 knockdown mitigated proinflammatory and oxidative injury in MRL/lpr mice. MRL/lpr mice were tail-intravenous injected with lenti-shHDAC3 or lenti-shNC. (**A**) Renal injury and inflammation were monitored using H&E staining. (**B**) IgG deposition was detected using IHC staining (**C**) Caspase3 protein expression was observed by IHC analysis. (**D**) Proinflammatory cytokines (IL-6 and IL-1β) and profibrotic cytokine (TGF-β) in kidney tissues were determined by qRT-PCR analysis. (**E**) Renal MDA and GSH levels and enzyme activities of SOD and CAT were assessed employing commercial kits. The results obtained are displayed as mean values±SD. The levels of significance were defined as follows: *p<0.05, **p<0.01 and ***p<0.001. CAT, catalase; GSH, glutathione; HDAC3, histone deacetylase 3; IHC, immunohistochemical; IL-6, interleukin-6; IL-1β, interleukin-1β; lenti-shHDAC3, lentiviral short hairpin RNA targeting HDAC3; lenti-shNC, lentiviral short hairpin RNA targeting negative control; MDA, malondialdehyde; MRL, Murphy Roths large; MRL/lpr, MRL/MpJ-Faslpr/J; qRT-PCR, quantitative real-time PCR; SOD, superoxide dismutase; TGF-β, transforming growth factor-β.

As shown in figure 3A, the renal tubule swelling and the excessive inflammatory cell infiltration in glomeruli were significantly alleviated by HDAC3 knockdown in MRL/lpr mice, compared with the lenti-shNC group, based on H&E staining. The renal IgG deposition in the lenti-shHDAC3 group was less than in the lenti-shNC group (figure 3B). Similarly, the expression of Caspase 3 protein in the kidney tissues of MRL/lpr mice was significantly downregulated following HDAC3 knockdown (figure 3C). Furthermore, levels of proinflammatory cytokines such as IL-6 and IL-1β, along with the profibrotic cytokine TGF-β in the kidney tissues of MRL/lpr mice subjected to lenti-shHDAC3 injection were markedly lower compared with those in the lenti-shNC group (figure 3D). Additionally, oxidative injury markers like MDA levels were reduced, while antioxidant indicators including GSH levels and activities of antioxidative enzymes SOD and CAT were enhanced due to HDAC3 knockdown in the kidney tissues of MRL/lpr mice (figure 3E). Overall, our data clarified that HDAC3 knockdown could alleviate the inflammatory and oxidative injury in the LN mice model.

### HDAC3 knockdown upregulated Nrf2 and alleviated ferroptosis in MRL/lpr mice

**Figure 4:**
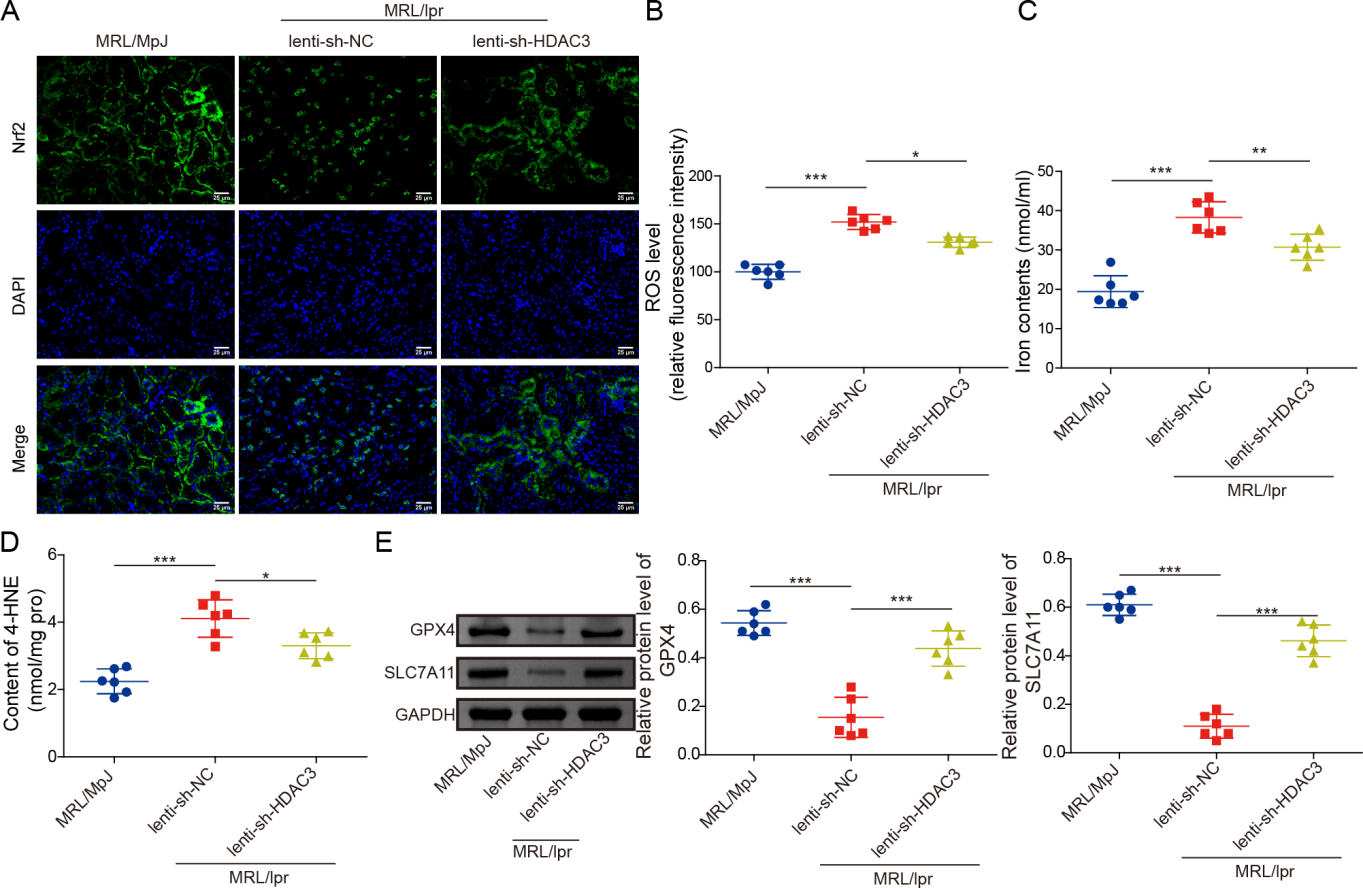
HDAC3 knockdown upregulated Nrf2 and alleviated ferroptosis in MRL/lpr mice. MRL/lpr mice were tail-intravenous injected with lenti-shHDAC3 or lenti-shNC. (**A**) Renal Nrf2 levels were evaluated by IF staining. (**B**) ROS levels were detected using commercial kits. (**C**) Iron ion contents were analysed by relevant kits. (**D**) 4-HNE contents were evaluated employing specific kits. (**E**) Renal GPX4 and SLC7A11 protein expressions were detected employing western blot analysis. The results obtained are displayed as mean values±SD. The levels of significance were defined as follows: *p<0.05, **p<0.01 and ***p<0.001. DAPI, 4’,6-diamidino-2-phenylindole; GAPDH, glyceraldehyde-3-phosphate dehydrogenase; GPX4, glutathione peroxidase 4; HDAC3, histone deacetylase 3; 4-HNE, 4-hydroxynonenal; IF, immunofluorescence; lenti-shHDAC3, lentiviral short hairpin RNA targeting HDAC3; lenti-shNC, lentiviral short hairpin RNA targeting negative control; MRL, Murphy Roths large; MRL/lpr, MRL/MpJ-Faslpr/J; Nrf2, nuclear factor erythroid 2-related factor 2; ROS, reactive oxygen species; SLC7A11, solute carrier family 7, (cationic amino acid transporter, y+ system) member 11.

To further explore the mechanism of HDAC3 in alleviating interstitial fibrosis, inflammation and oxidative injury, we analysed the expression levels of core oxidative modulators, including Nrf2 and GPX4, in serum and kidney tissues of patients with SLE and LN. Compared with the control group, the levels and mRNA expression densities of Nrf2/GPX4 were significantly decreased in the serum and kidney samples, and the lowest levels were identified in the LN group (online supplemental figure S1A,B). Moreover, the correlation analysis revealed that renal Nrf2 or GPX4 expression levels in the LN group were negatively correlated with SLEDAI Scores (online supplemental figure S1C). Additionally, those levels of Nrf2 or GPX4 in kidney tissues of the LN group were also negatively correlated with the HDAC3 expression levels (online supplemental figure S1D). In line with that, HDAC3 knockdown significantly upregulated the Nrf2 expression in the kidney tissues of MRL/lpr mice, compared with the mice with lenti-shNC injection (figure 4A). HDAC3 inhibition also markedly decreased the renal ROS accumulation levels in contrast to the NC group (figure 4B). As for renal ferroptosis, the levels of ferroptosis markers such as iron ion and 4-HNE in kidney tissues of MRL/lpr mice were upregulated, while that phenomenon was reversed by the knockdown of HDAC3 (figure 4C,D). Furthermore, the renal ferroptosis repressive proteins, including GPX4 and SLC7A11 levels, were decreased in MRL/lpr mice, whereas the knockdown of HDAC3 significantly enhanced those protein expressions (figure 4E). In sum, this part of the results elaborated that the knockdown of HDAC3 could promote Nrf2 expression and alleviate ferroptosis in the kidney tissues of MRL/lpr mice.

### HDAC3 inhibition reversed the cell viability decrease, proinflammatory cytokine increase and fibrosis enhancement in the LN-serum-induced HK-2 cells

**Figure 5:**
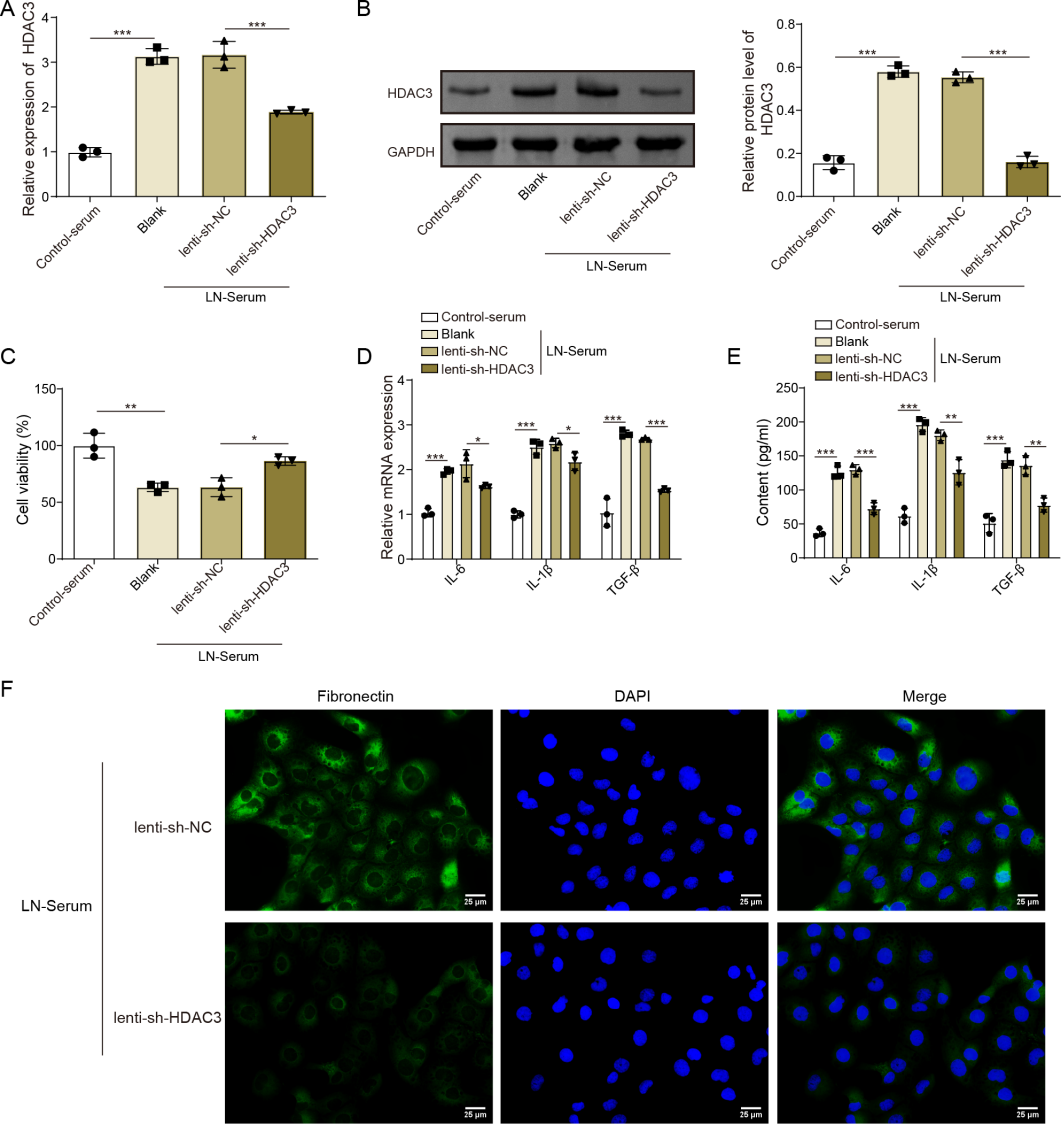
HDAC3 inhibition reversed the cell viability inhibition, proinflammatory cytokine elevation and fibrosis enhancement in LN-serum-induced HK-2 cells. HK-2 cells were stimulated with Blank-serum or LN-serum and then infected with lenti-shHDAC3 or lenti-shNC. (**A**) HDAC3 gene expression was determined by qRT-PCR analysis. (**B**) HDAC3 protein expression was detected employing western blot analysis. (**C**) Cell viability was assessed by CCK-8 kits. (**D**) Proinflammatory cytokines (IL-6 and IL-1β) and profibrotic cytokine (TGF-β) gene expressions were determined by qRT-PCR analysis. (**E**) Proinflammatory cytokines (IL-6 and IL-1β) and profibrotic cytokine (TGF-β) levels were evaluated using ELISA kits. (**F**) Fibronectin levels were assessed by IF staining. The results obtained are displayed as mean values±SD, including a sample size of three for each group. The levels of significance were defined as follows: *p<0.05, **p<0.01 and ***p<0.001. CCK-8, Cell Counting Kit-8; DAPI, 4’,6-diamidino-2-phenylindole; ELISA, enzyme-linked immunosorbent assay; GAPDH, glyceraldehyde-3-phosphate dehydrogenase; HDAC3, histone deacetylase 3; HK-2 cells, human renal proximal tubular epithelial cells; IF, immunofluorescence; IL-6, interleukin-6; IL-1β, interleukin-1β; lenti-shHDAC3, lentiviral short hairpin RNA targeting HDAC3; lenti-shNC, lentiviral short hairpin RNA targeting negative control; LN, lupus nephritis; qRT-PCR, quantitative real-time PCR; TGF-β, transforming growth factor-β.

To demonstrate the deep mechanism of HDAC3 in LN, the LN cellular model was conducted using the HK-2 cells stimulated with serum of patients with LN, and the LN-serum-treated cells were infected with lenti-shHDAC3 or lenti-shNC. As displayed in figure 5A,B, LN-serum remarkably enhanced HDAC3 mRNA and protein expression in HK-2 cells, while that was reversed by HDAC3 silencing. Further, LN-serum significantly downregulated the cell viability, while the LN-serum-decreased cell viability was abolished by HDAC3 knockdown in HK-2 cells (figure 5C). In addition, LN-serum markedly boosted the inflammatory cytokine (IL-1β, IL-6) and profibrotic factor (TGF-β); at the same time, the co-treatment of HDAC3 inhibition reversed that LN-serum-modulated trend (figure 5D,E). Similarly, LN-serum promoted the fibronectin protein expression, while that was also abolished by HDAC3 knockdown (figure 5F). In conclusion, our data clarified that in LN-serum exposed-HK-2 cells, decreased cell viability, increased proinflammatory cytokines and excessive fibrotic phenotype were abolished by HDAC3 knockdown.

### HDAC3 knockdown abrogated the elevated ferroptosis in LN-serum-exposed HK-2 cells

**Figure 6:**
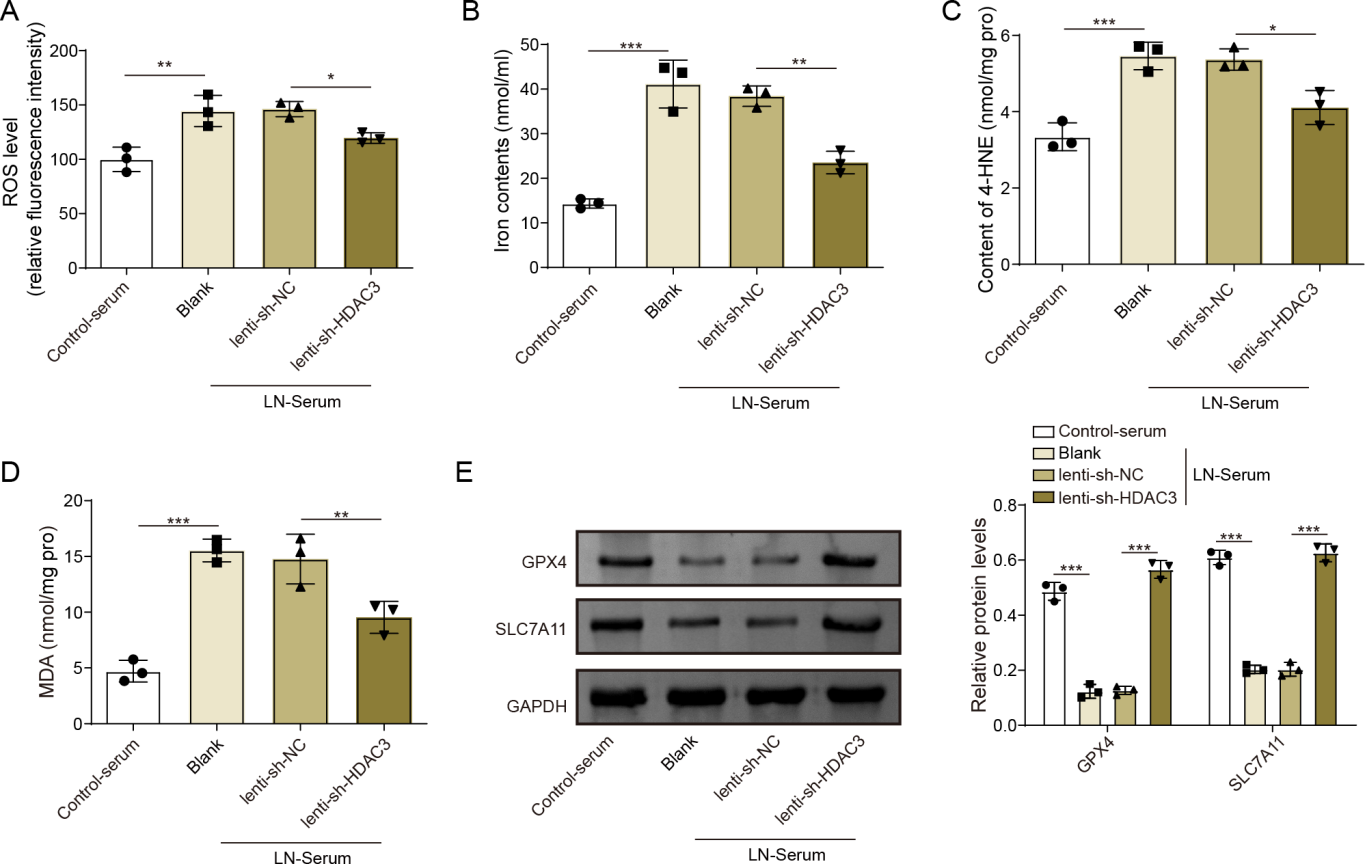
HDAC3 knockdown abrogated the elevated ferroptosis in LN-serum exposed-HK2 cells. HK-2 cells were stimulated with Blank-serum or LN-serum and then infected with lenti-shHDAC3 or lenti-shNC. (**A**) ROS levels were detected using commercial kits. (**B**) Iron ion contents were analysed using relevant kits. (**C**) 4-HNE contents were evaluated employing specific kits. (**D**) MDA levels were analysed using relevant kits. (**E**) GPX4 and SLC7A11 protein expressions were detected employing western blot analysis. The results obtained are displayed as mean values±SD, including a sample size of three for each group. The levels of significance were defined as follows: *p<0.05, **p<0.01 and ***p<0.001. GAPDH, glyceraldehyde-3-phosphate dehydrogenase; GPX4, glutathione peroxidase 4; HDAC3, histone deacetylase 3; HK-2 cells, human renal proximal tubular epithelial cells; 4-HNE, 4-hydroxynonenal; lenti-shHDAC3, lentiviral short hairpin RNA targeting HDAC3; lenti-shNC, lentiviral short hairpin RNA targeting negative control; LN, lupus nephritis; MDA, malondialdehyde; ROS, reactive oxygen species; SLC7A11, solute carrier family 7, (cationic amino acid transporter, y+ system) member 11.

As demonstrated in figure 6A, HDAC3 inhibition in HK-2 cells alleviated the LN-serum-induced increase of ROS levels. LN-serum also elevated the levels of ferroptosis markers such as iron ion and 4-HNE, while that was reversed by the HDAC3 (figure 6B,C). Moreover, HDAC3 knockdown also abolished the LN-serum-increased oxidative stress marker MDA level in HK-2 cells (figure 6D). Consistently, LN-serum significantly upregulated the protein expressions of GPX4 and SLC7A11, whereas the co-treatment of HDAC3 inhibition overturned that (figure 6E). Overall, our results revealed that HDAC3 knockdown ameliorated ferroptosis in LN-serum-exposed HK-2 cells.

### HDAC3 inhibition disrupted Keap1-Nrf2 interaction to trigger Nrf2 activation

**Figure 7:**
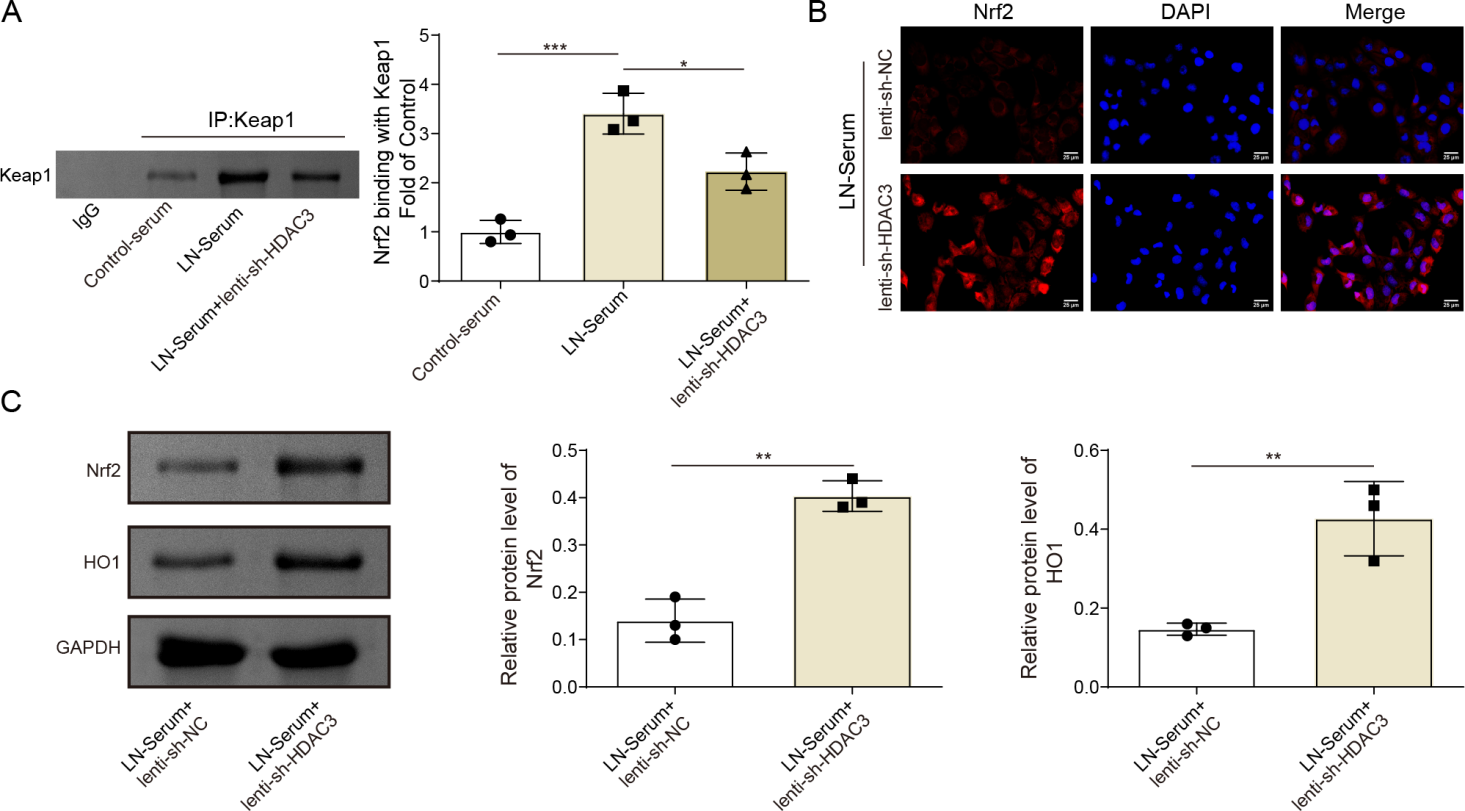
HDAC3 inhibition disrupted Keap1-Nrf2 interaction to trigger Nrf2 activation. HK-2 cells were stimulated with Blank-serum or LN-serum and then infected with lenti-shHDAC3. (**A**) Co-IP analysis analysed the interaction between Nrf2 and Keap1. HK-2 cells were stimulated with LN-serum and then infected with lenti-shNC or lenti-shHDAC3 (**B–C**). (**B**) Nrf2 levels were evaluated by IF staining. (**C**) Nrf2 and HO-1 protein expressions were detected employing western blot analysis. The results obtained are displayed as mean values±SD, including a sample size of three for each group. The levels of significance were defined as follows: *p<0.05, **p<0.01 and ***p<0.001. Co-IP, co-immunoprecipitation; DAPI, 4’,6-diamidino-2-phenylindole; GAPDH, glyceraldehyde-3-phosphate dehydrogenase; HDAC3, histone deacetylase 3; HK-2 cells, human renal proximal tubular epithelial cells; HO-1, heme oxygenase 1; IF, immunofluorescence; Keap1, Kelch-like ECH-associated protein 1; lenti-shHDAC3, lentiviral short hairpin RNA targeting HDAC3; lenti-shNC, lentiviral short hairpin RNA targeting negative control; LN, lupus nephritis; Nrf2, nuclear factor erythroid 2-related factor 2.

LN-serum remarkably promoted the interaction between Nrf2 and Keap1, while the co-treated HDAC3 inhibition reversed the LN-serum-regulated enhancement of Nrf2 binding with Keap1 in HK-2 cells (figure 7A). In line with that, HDAC3 knockdown significantly enhanced the Nrf2 nuclear translocation in LN-serum-treated HK-2 cells (figure 7B). Under LN serum stimulation, HDAC3 inhibition markedly upregulated Nrf2 and HO-1 protein expression in HK-2 cells (figure 7C). Collectively, these data demonstrated that HDAC3 inhibition could disrupt Keap1-Nrf2 interaction to trigger Nrf2 activation in LN-serum-induced HK-2 cells.

## Discussion

LN is ranked as the most prevalent complication of SLE, with extremely high morbidity and mortality.^30^ Around 40% of individuals diagnosed with SLE go on to experience LN, while it is estimated that about 10% of those with LN will progress to ESRD within a decade.^31^ It is urgent to find effective targets and pathways to supplement the current therapies, such as agents that suppress abnormal immunological reactions (eg, belimumab), besides hydroxychloroquine.^32^ RIF is regarded as the principal change in the pathogenetic process of LN, and RIF and tubular atrophy are positively associated with the renal survival of patients with LN.^33^ Abnormal renal inflammation, oxidative stress and ferroptosis are contended as the primary pathological basis of RIF, so the reagents inhibiting them are explored to relieve RIF, such as puerarin and salidroside.^15 34^ In our study, we identified that HDAC3 was significantly increased in the serum and kidney tissues of patients with LN, and its depletion could inhibit the RIF, inflammation, oxidative stress and ferroptosis via promoting Nrf2 in the MRL/lpr female mice and LN-serum-stimulated HK-2 cells, the animal and cellular LN models. These findings deepened the understanding of pathological mechanisms of RIF in LN and evidenced the role of the HDAC3/Nrf2 axis in the diagnosis and intervention of LN.

As classic epigenetic proteins, HDACs are tightly involved in the regulation of renal normal function and kidney tissue health.^35^ HDAC3 is a vital member of the HDAC family, and it also engages with the potent effects on renal protection under disease and injury conditions.^18 36^ HDAC3 expression is induced in the mice and cell models of kidney fibrosis disease, and its inhibition can ameliorate the fibrosis changes.^20 37^ In the serum and kidney tissues of patients with LN, we first identified that HDAC3 expressions were significantly increased, and those in the LN group posed the highest level compared with healthy people and patients with SLE, and HDAC3 expression levels were positively correlated with the total SLEDAI Scores of LN. Those observations also highlighted the significance of HDAC3 in the progression of LN for the first time. In our previous study, the inhibition of HDAC3 has restrained the hyperuricaemia-induced RIF development.^21^ Consistently, HDAC3 inhibition also attenuated RIF in the MRL/lpr mice, which enriched its role in kidney disease protection. Moreover, HDAC3 knockdown markedly alleviated the RIF-accompanied inflammatory injury of the LN mice model. That was in accordance with the corresponding role of HDAC3 and other HDAC proteins in renal fibrosis models.^20 38^ Innovatively, we put forward that HDAC3 inhibition reduced the RIF-relevant oxidative injury, deepening the modulatory mechanism of HDAC3 in RIF repression. In the cell model, HADC3 knockdown also upregulated the cell viability and downregulated the fibrosis phenotype (fibronectin protein marker) and inflammatory cytokine release in the LN-serum-exposed renal proximal tubular epithelial cells. It validated the function of HDAC3 inhibition in renal fibrosis and inflammation at the cell level. Therefore, we first clarified the role of HDAC3 inhibition in the RIF suppression of LN, offering a potentially precise inhibitor of LN therapy.

Nrf2 is a classic transcription factor of cellular oxidative stress reactions and is a vital modulator of cell redox homoeostasis maintenance.^39^ During renal fibrosis, Nrf2 can regulate the antioxidant and cytoprotective gene expression to alleviate the fibrosis progression and severity as an underlying therapeutic target.^40^ Further, the excessive accumulation of lipid peroxide accompanied by iron deposition always induces ferroptosis, which is also mediated by Nrf2.^41^ Ferroptosis refers to a cell death pathway driven by lipid peroxidation and reliant on iron, which has emerged as a crucial focus in LN pathogenesis.^42^ The activation of the Nrf2/GPX4/SLC7A11 axis poses a significant effect on the inhibition of ferroptosis.^43^ In this study, we found that the Nrf2/GPX4 levels were downregulated in the serum and kidney tissues of patients with LN and their renal levels were negatively correlated with the total SLEDAI Scores. These results were consistently kept in line with the relevant studies of the role of Nrf2 in constraining LN.^44^ Moreover, we originally identified that renal Nrf2/GPX4 levels were negatively correlated with HDAC3 in patients with LN, which was partially similar to the interaction of Nrf2/HDAC3 in the peritoneal fibrosis model.^22^ The silenced HDAC3 markedly upregulated the ferroptosis inhibitive proteins of the Nrf2/GPX4/SLC7A11 expressions and downregulated oxidative stress marker levels in the MRL/lpr mice and LN-serum-induced HK-2 cells. The clarified mechanism of HDAC3 in ferroptosis of the LN model was in keeping with the evidence in the acute kidney injury progression to the chronic kidney disease mice model.^36^ Under steady-state conditions, Keap1 is part of an E3 ubiquitin ligase that tightly controls Nrf2 activity.^45^ In reaction to stress, a complex molecular mechanism is activated, driven by sensor cysteines located within the KEAP1 protein, which mechanism enables Nrf2 to evade the process of ubiquitination, thereby allowing it to accumulate within the cell and translocate to the nucleus, where it plays a crucial role in initiating and promoting its antioxidant transcription function.^46^ In the LN cell model, we first revealed that HDAC3 inhibition disrupted the Keap1-Nrf2 interaction to trigger Nrf2 activation, which was like the finding in an endothelial model of diabetes mellitus.^47^ So, we demonstrated that HDAC3 inhibition could repress ferroptosis via activation of Nrf2 in the LN models.

Collectively, this study clarified that HDAC3 inhibition could repress RIF, oxidative stress, inflammation and ferroptosis by upregulating Nrf2 in the mice and cell models of LN. It offered precise evidence that the HDAC3/Nrf2 axis could be employed as underlying targets of LN therapy in the future. Nevertheless, the validation of this mechanism in clinical samples is still necessary. Furthermore, there was a higher prevalence of thrombocytopenia among patients with LN. Therefore, exploring the detailed prevalence of antiphospholipid antibodies and related manifestations in subsequent studies is warranted. In addition, HDAC3 expression was positively correlated with the total SLEDAI of patients with LN, while the correlation with the renal domains of SLEDAI and histological measures as the activity and chronicity index also needs further exploration. Next, we used the serum derived from patients with LN to treat HK-2 cells for establishing the in vitro model, which was like the previous study.^48^ The mechanisms by which serum-induced decreases in cell viability and increases in fibrotic activity, as well as the alleviation mediated by HDAC3 knockdown, are regulated by antibodies or cytokines remain unclear, as the limitation of the present study, and warrant subsequent investigation in future studies. In conclusion, our research uncovered the role and mechanism of HDAC3 in RIF suppression, which could serve as the target for medicine development related to LN.

## Supplementary material

10.1136/lupus-2025-001666 online supplemental file 1

10.1136/lupus-2025-001666 online supplemental file 2

## Data Availability

Data are available upon reasonable request.

## References

[1] Zucchi D, Elefante E, Schilirò D (2022). One year in review 2022: systemic lupus erythematosus. Clin Exp Rheumatol.

[2] Alforaih N, Whittall-Garcia L, Touma Z (2022). A Review of Lupus Nephritis. J Appl Lab Med.

[3] Lamba P, Nam KH, Contractor J (2020). Nephritic Syndrome. Prim Care.

[4] Parikh SV, Almaani S, Brodsky S (2020). Update on Lupus Nephritis: Core Curriculum 2020. Am J Kidney Dis.

[5] Shin JI, Li H, Park S (2022). Induction and Maintenance Treatment of Lupus Nephritis: A Comprehensive Review of Meta-Analyses. J Clin Med.

[6] Houssiau FA, Vasconcelos C, D’Cruz D (2002). Immunosuppressive therapy in lupus nephritis: the Euro-Lupus Nephritis Trial, a randomized trial of low-dose versus high-dose intravenous cyclophosphamide. Arthritis Rheum.

[7] Ponticelli C, Moroni G (2017). Hydroxychloroquine in systemic lupus erythematosus (SLE). Expert Opin Drug Saf.

[8] Yu C, Li P, Dang X (2022). Lupus nephritis: new progress in diagnosis and treatment. J Autoimmun.

[9] Sciascia S, Cozzi M, Barinotti A (2022). Renal Fibrosis in Lupus Nephritis. Int J Mol Sci.

[10] Wang S, Wu M, Chiriboga L (2022). Membrane attack complex (MAC) deposition in renal tubules is associated with interstitial fibrosis and tubular atrophy: a pilot study. Lupus Sci Med.

[11] Sun Y-S, Huang D-F, Chang F-P (2024). Interstitial fibrosis increases the risk of end-stage kidney disease in patients with lupus nephritis. Rheumatology (Oxford).

[12] Zhao J, Meng M, Zhang J (2019). Astaxanthin ameliorates renal interstitial fibrosis and peritubular capillary rarefaction in unilateral ureteral obstruction. Mol Med Rep.

[13] Du L, Chen Y, Shi J (2023). Inhibition of S100A8/A9 ameliorates renal interstitial fibrosis in diabetic nephropathy. Metab Clin Exp.

[14] Nady ME, Abd El-Raouf OM, El-Sayed E-SM (2024). Linagliptin Mitigates TGF-β1 Mediated Epithelial-Mesenchymal Transition in Tacrolimus-Induced Renal Interstitial Fibrosis via Smad/ERK/P38 and HIF-1α/LOXL2 Signaling Pathways. Biol Pharm Bull.

[15] Li R, Guo Y, Zhang Y (2019). Salidroside Ameliorates Renal Interstitial Fibrosis by Inhibiting the TLR4/NF-κB and MAPK Signaling Pathways. IJMS.

[16] Hong S, Lu Y (2013). Omega-3 fatty acid-derived resolvins and protectins in inflammation resolution and leukocyte functions: targeting novel lipid mediator pathways in mitigation of acute kidney injury. Front Immunol.

[17] Xiong W, Xiong Z, Song A (2023). UCP1 alleviates renal interstitial fibrosis progression through oxidative stress pathway mediated by SIRT3 protein stability. J Transl Med.

[18] Zhang L, Cao W (2022). Histone deacetylase 3 (HDAC3) as an important epigenetic regulator of kidney diseases. *J Mol Med (Berl*).

[19] He R, Liu B, Geng B (2023). The role of HDAC3 and its inhibitors in regulation of oxidative stress and chronic diseases. Cell Death Discov.

[20] Wang Y, Jiao B, Hu Z (2024). Critical Role of histone deacetylase 3 in the regulation of kidney inflammation and fibrosis. Kidney Int.

[21] Hu L, Yang K, Mai X (2022). Depleted HDAC3 attenuates hyperuricemia-induced renal interstitial fibrosis via miR-19b-3p/SF3B3 axis. Cell Cycle.

[22] Kim JE, Han D, Kim KH (2024). Protective effect of Cyclo(His-Pro) on peritoneal fibrosis through regulation of HDAC3 expression. FASEB J.

[23] Mahmoud GA, Zayed HS, Ghoniem SA (2015). Renal outcomes among Egyptian lupus nephritis patients: a retrospective analysis of 135 cases from a single centre. Lupus (Los Angel).

[24] Zheng C-Z, Yan W-W, Luo Y-L (2020). Value of sTNF-R1 and linc0597 as indicators for disease activity and diagnosis of lupus nephritis. Eur Rev Med Pharmacol Sci.

[25] Zhao W, Wu C, Li LJ (2018). RNAi Silencing of HIF-1α Ameliorates Lupus Development in MRL/lpr Mice. Inflammation.

[26] Liu CJ, Tang SJ, Chou CC (2020). *In Vivo* Suppression of Autophagy via Lentiviral shRNA Targeting Atg5 Improves Lupus-Like Syndrome. Biomed Res Int.

[27] Wen Y, Zhang X, Wei L (2023). Gastrodin attenuates renal injury and collagen deposition *via* suppression of the TGF-β1/Smad2/3 signaling pathway based on network pharmacology analysis. Front Pharmacol.

[28] Xue L, Zhang Y, Xu J (2021). Anti-TWEAK Antibody Alleviates Renal Interstitial Fibrosis by Increasing PGC-1α Expression in Lupus Nephritis. J Inflamm Res.

[29] Wang J, Chen Q, Zhang Z (2021). Azithromycin alleviates systemic lupus erythematosus via the promotion of M2 polarisation in lupus mice. Cell Death Discov.

[30] Liu C, Gan Y, Yong W (2024). OTUB1 regulation of ferroptosis and the protective role of ferrostatin-1 in lupus nephritis. Cell Death Dis.

[31] Siegel CH, Sammaritano LR (2024). Systemic Lupus Erythematosus: A Review. JAMA.

[32] Furie R, Rovin BH, Houssiau F (2020). Two-Year, Randomized, Controlled Trial of Belimumab in Lupus Nephritis. N Engl J Med.

[33] Wilson PC, Kashgarian M, Moeckel G (2018). Interstitial inflammation and interstitial fibrosis and tubular atrophy predict renal survival in lupus nephritis. Clin Kidney J.

[34] Jian J, Wang D, Xiong Y (2023). Puerarin alleviated oxidative stress and ferroptosis during renal fibrosis induced by ischemia/reperfusion injury via TLR4/Nox4 pathway in rats. Acta Cir Bras.

[35] Brilli LL, Swanhart LM, de Caestecker MP (2013). HDAC inhibitors in kidney development and disease. Pediatr Nephrol.

[36] Zhang L, Chen F, Dong J (2023). HDAC3 aberration-incurred GPX4 suppression drives renal ferroptosis and AKI-CKD progression. Redox Biol.

[37] Zhang X, Wang J, Xiang S (2024). Astragaloside I from Astragalus Attenuates Diabetic Kidney Disease by Regulating HDAC3/Klotho/TGF-beta1 Loop. Am J Chin Med.

[38] Kumar P, Gogulamudi VR, Periasamy R (2017). Inhibition of HDAC enhances STAT acetylation, blocks NF-κB, and suppresses the renal inflammation and fibrosis in *Npr1* haplotype male mice. Am J Physiol Renal Physiol.

[39] Yamamoto M, Kensler TW, Motohashi H (2018). The KEAP1-NRF2 System: a Thiol-Based Sensor-Effector Apparatus for Maintaining Redox Homeostasis. Physiol Rev.

[40] Hassanein EHM, Ibrahim IM, Abd-Alhameed EK (2023). Nrf2/HO-1 as a therapeutic target in renal fibrosis. Life Sci.

[41] Dodson M, Castro-Portuguez R, Zhang DD (2019). NRF2 plays a critical role in mitigating lipid peroxidation and ferroptosis. Redox Biol.

[42] Morel L, Scindia Y (2024). Functional consequence of Iron dyshomeostasis and ferroptosis in systemic lupus erythematosus and lupus nephritis. Clin Immunol.

[43] Yuan Y, Zhai Y, Chen J (2021). Kaempferol Ameliorates Oxygen-Glucose Deprivation/Reoxygenation-Induced Neuronal Ferroptosis by Activating Nrf2/SLC7A11/GPX4 Axis. Biomolecules.

[44] Ebihara S, Tajima H, Ono M (2016). Nuclear factor erythroid 2-related factor 2 is a critical target for the treatment of glucocorticoid-resistant lupus nephritis. Arthritis Res Ther.

[45] Bellezza I, Giambanco I, Minelli A (2018). Nrf2-Keap1 signaling in oxidative and reductive stress. Biochim Biophys Acta Mol Cell Res.

[46] Baird L, Yamamoto M (2020). The Molecular Mechanisms Regulating the KEAP1-NRF2 Pathway. Mol Cell Biol.

[47] Huang S, Chen G, Sun J (2021). Histone deacetylase 3 inhibition alleviates type 2 diabetes mellitus-induced endothelial dysfunction via Nrf2. Cell Commun Signal.

[48] Zou Y, Wang D, Sun W (2024). Fibroblast growth factor 21 mitigates lupus nephritis progression via the FGF21/Irgm 1/NLRP3 inflammasome pathway. Int Immunopharmacol.

