# Supplementary material for: HDAC3 knockdown inhibits ferroptosis via upregulating Nrf2 to alleviate renal interstitial fibrosis in lupus nephritis
Source: Lupus Sci Med. 2025 Oct 22;12(2):e001666. doi: 10.1136/lupus-2025-001666 (PMC12551480; doi:10.1136/lupus-2025-001666)
Supplement: online supplemental file 1 [file lupus-12-2-s001.docx]

**Supplementary Materials**

**Table S1. Analysis of general data of subjects in the three groups.**

| **Indicators** | **The control group** | **The LN group** | **The SLE group** | **X^2^/F/t** | **P** |
| --- | --- | --- | --- | --- | --- |
| Gender |  |  |  | 1.577 | 0.454 |
| Male | 13 | 10 | 9 |  |  |
| Female | 9 | 12 | 13 |  |  |
| Age (year) | 34.55±7.42 | 29.82±3.10 | 33.09±4.87 | 4.381 | <0.05 |
| Course of disease (month) | - | 12.68±1.43 | 12.64±1.26 | 0.112 | 0.911 |
| BMI (kg/m^2^) | 24.03±1.63 | 23.19±1.90 | 23.04±1.66 | 2.060 | 0.136 |
| Dietary preference |  |  |  | 3.397 | 0.183 |
| Light | 14 | 12 | 8 |  |  |
| Spick | 8 | 10 | 14 |  |  |
| Smoking history |  |  |  | 1.234 | 0.539 |
| Yes | 13 | 15 | 10 |  |  |
| No | 9 | 7 | 12 |  |  |
| Educational level |  |  |  | 1.735 | 0.420 |
| ≥ high school | 10 | 6 | 7 |  |  |
| < high school | 12 | 16 | 15 |  |  |
| Alcohol abuse |  |  |  | 2.632 | 0.268 |
| Yes | 11 | 12 | 16 |  |  |
| No | 11 | 10 | 6 |  |  |

**Table S2. Comparison of clinical symptoms and pathological parameters between the LN group and SLE group (n), (**$\overline{\boldsymbol{x}}$**±sd).**

| **Indicators** | **The LN group (n=22)** | **The SLE group (n=22)** | **X^2^/t** | **P value** |
| --- | --- | --- | --- | --- |
| Fever |  |  | 6.286 | <0.05 |
| Yes | 12 | 4 |  |  |
| No | 10 | 18 |  |  |
| Rash |  |  | 0.376 | 0.540 |
| Yes | 8 | 10 |  |  |
| No | 14 | 12 |  |  |
| Arthralgia |  |  | 4.125 | <0.05 |
| Yes | 9 | 3 |  |  |
| No | 13 | 19 |  |  |
| Serum uric acid (mmol/L) | 0.50±0.13 | 0.32±0.07 | 6.051 | <0.001 |
| Serum creatinine (mg/dL) | 0.78±0.20 | 0.70±0.19 | 1.245 | 0.220 |
| Leukopenia |  |  | 0.834 | 0.361 |
| Positive | 11 | 14 |  |  |
| Negative | 11 | 8 |  |  |
| Anemia |  |  | 0.820 | 0.365 |
| Yes | 10 | 13 |  |  |
| No | 12 | 9 |  |  |
| Erythrocytopenia |  |  | 0.820 | 0.365 |
| Positive | 13 | 10 |  |  |
| Negative | 9 | 12 |  |  |
| Thrombocytopenia |  |  | 11.208 | <0.001 |
| Positive | 15 | 4 |  |  |
| Negative | 7 | 18 |  |  |
| Decreased complement C3 |  |  | 5.867 | <0.05 |
| Positive | 14 | 6 |  |  |
| Negative | 8 | 16 |  |  |
| Decreased complement C4 |  |  | 2.277 | 0.131 |
| Positive | 14 | 9 |  |  |
| Negative | 8 | 13 |  |  |
| ANA |  |  | 17.111 | 0.007 |
| Positive | 20 | 18 |  |  |
| Negative | 2 | 4 |  |  |
| ANA homogeneous type |  |  | 13.211 | <0.001 |
| Positive | 12 | 1 |  |  |
| Negative | 10 | 21 |  |  |
| ANA peripheral type |  |  | 5.939 | <0.05 |
| Positive | 9 | 2 |  |  |
| Negative | 13 | 20 |  |  |
| ANA spot type |  |  | 1.467 | 0.226 |
| Positive | 14 | 10 |  |  |
| Negative | 8 | 12 |  |  |
| ANA nucleolar type |  |  | 0.863 | 0.353 |
| Positive | 15 | 12 |  |  |
| Negative | 7 | 10 |  |  |
| Anti-ds-DNA antibody |  |  | 6.907 | <0.05 |
| Positive | 19 | 10 |  |  |
| Negative | 3 | 12 |  |  |
| Anti SM antibody |  |  | 5.939 | <0.05 |
| Positive | 2 | 9 |  |  |
| Negative | 20 | 13 |  |  |
| ANCA |  |  | 5.500 | <0.05 |
| Positive | 7 | 1 |  |  |
| Negative | 15 | 21 |  |  |

**Table S3. Demographic and clinical characteristics of SLE patients.**

| **No./Sex** | **SLEDAI score** | **Proteinuria**  **(mg/24h)** | **Clinical manifestations** |
| --- | --- | --- | --- |
| LN1/F | 17.0 | 970 | Nephritis, cytopenia, polyserositis. |
| LN2/F | 7.0 | 1170 | Nephritis, arthralgia, skin vasculitis. |
| LN3/F | 11.0 | 800 | Nephritis, cytopenia. |
| LN4/F | 18.0 | 1130 | Nephritis, skin vasculitis. |
| LN5/F | 16.0 | 580 | Nephritis. |
| LN6/F | 11.0 | 1890 | Nephritis, cytopenia, polyserositis, skin vasculitis |
| LN7/F | 7.0 | 920 | Nephritis, cytopenia. |
| LN8/F | 13.0 | 1060 | Cytopenia |
| LN9/F | 7.0 | 5040 | Arthralgia, cytopenia, polyserositis, skin vasculitis. |
| LN10/F | 15.0 | 850 | Nephritis, cytopenia, polyserositis |
| LN11/F | 10.0 | 2210 | Nephritis, cytopenia. |
| LN12/F | 8.0 | 1070 | Cytopenia, nephritis. |
| LN13//F | 13.0 | 680 | Nephritis. |
| LN14/M | 9.0 | 920 | Nephritis, cytopenia. |
| LN15/M | 10.0 | 1220 | Nephritis, arthralgia, skin vasculitis. |
| LN16/M | 10.0 | 480 | Arthralgia, cytopenia, polyserositis, skin vasculitis. |
| LN17/M | 10.0 | 1170 | Arthralgia, cytopenia. |
| LN18/M | 17.0 | 890 | Nephritis, cytopenia, skin vasculitis. |
| LN19/M | 13.0 | 1800 | Nephritis, cytopenia, cytopenia  neuropsychiatric involvement. |
| LN20/M | 8.0 | 1110 | Nephritis, skin vasculitis. |
| LN21/M | 11.0 | 580 | Nephritis, cytopenia, polyserositis. |
| LN22/M | 9.0 | 1220 | Nephritis, polyserositis, skin vasculitis. |

LN: lupus nephritis; SLEDAI: SLE disease activity index; M: male; F: female.

**Table S4. Effects of HDAC3 knockdown on bodv weight, organ index, blood counting and renal function after 10-week administration.**

| **Indicators** | **MRL/MpJ** | **MRL/Lpr** | |
| --- | --- | --- | --- |
|  |  | Lenti-sh-NC | Lenti-sh-HDAC3 |
| N | 6 | 6 | 6 |
| Body weight (g) | 32.9±1.54 | 28.8±1.73^##^ | 27.6±1.60 |
| Anti-dsDNA (μg/ml) | 0.55±0.03 | 3.58±0.16^###^ | 1.55±0.10^***^ |
| Kidney weight (g) | 0.22±0.008 | 0.40±0.029^##^ | 0.24±0.036^**^ |
| Kidney index (10^-3^) | 6.1±0.06 | 12.9±0.90^###^ | 8.8±0.82^*^ |
| Spleen weight (g) | 0.060±0.007 | 0.094±0.004^##^ | 0.087±0.011^*^ |
| Spleen index (10^-3^) | 1.8±0.13 | 3.8±0.31^###^ | 3.2±0.29^*^ |
| WBC (10^9^/L) | 11.5±1.06 | 16.2±1.41^###^ | 12.6±0.42^***^ |
| Lymphocyte (10^9^/L) | 2.07±0.37 | 3.95±0.64^###^ | 2.64±0.32^*^ |
| Monocyte (10^9^/L) | 0.26±0.11 | 0.33±0.05 | 0.30±0.08 |
| Neutrophil (10^9^/L) | 8.86±0.77 | 9.66±0.62 | 8.93±0.42 |
| Eosinophils (10^9^/L) | 0.00±0.00 | 0.32±0.10^###^ | 0.09±0.02^***^ |
| μg Ualb/mg creatinine | 2.5±0.16 | 32.1±2.47^###^ | 17.0±1.35^***^ |
| BUN (mg/dL) | 23.0±2.25 | 46.6±3.23^###^ | 28.7±2.44^***^ |
| Scr (mg/dL) | 2.35±0.19 | 3.64±0.18^###^ | 2.71±0.12^**^ |
| Ratio of BUN/Scr | 9.82±1.01 | 12.84±1.03^###^ | 10.58±0.85^**^ |

# represent the comparison between MRL/MpJ group and MRL/Lpr+Lenti-sh-NC group. * represent the comparison between MRL/Lpr+Lenti-sh-NC group and MRL/Lpr+Lenti-sh-HDAC3 group.

**Table S5. The primers for quantitative real-time polymerase chain reaction assay.**

| Gene | Primer sequences (5’-3’) |
| --- | --- |
| *HDAC3* | F: GAGTTCTGCTCGCGTTACACAG  R: CGTTGACATAGCAGAAGCCAGAG |
| *COL-1* | F: GATTCCCTGGACCTAAAGGTGC  R: AGCCTCTCCATCTTTGCCAGCA |
| *Fibronectin* | F: ACAACACCGAGGTGACTGAGAC  R: GGACACAACGATGCTTCCTGAG |
| *IL-6* | F: AGACAGCCACTCACCTCTTCAG  R: TTCTGCCAGTGCCTCTTTGCTG |
| *IL-1β* | F: CCACAGACCTTCCAGGAGAATG  R: GTGCAGTTCAGTGATCGTACAGG |
| *TGF-1β* | F: TACCTGAACCCGTGTTGCTCTC  R: GTTGCTGAGGTATCGCCAGGAA |
| *Nrf2* | F: CACATCCAGTCAGAAACCAGTGG  R: GGAATGTCTGCGCCAAAAGCTG |
| *GPX4* | F: ACAAGAACGGCTGCGTGGTGAA  R: GCCACACACTTGTGGAGCTAGA |
| *GAPDH* | F: CTCCTTTTTGGCAGACACTGGTG  R: ATGCCAGTGAGCTTCCCGTTCAG |

*HDAC3*, histone deacetylase 3; *COL-1*, collagen type I; *IL-6*, interleukin 6; *IL-1β*, interleukin 1β; *TGF-1β*, transforming growth factor 1β; *Nrf2*, nuclear factor erythroid 2-related factor 2; *GPX4,* glutathione peroxidase 4.


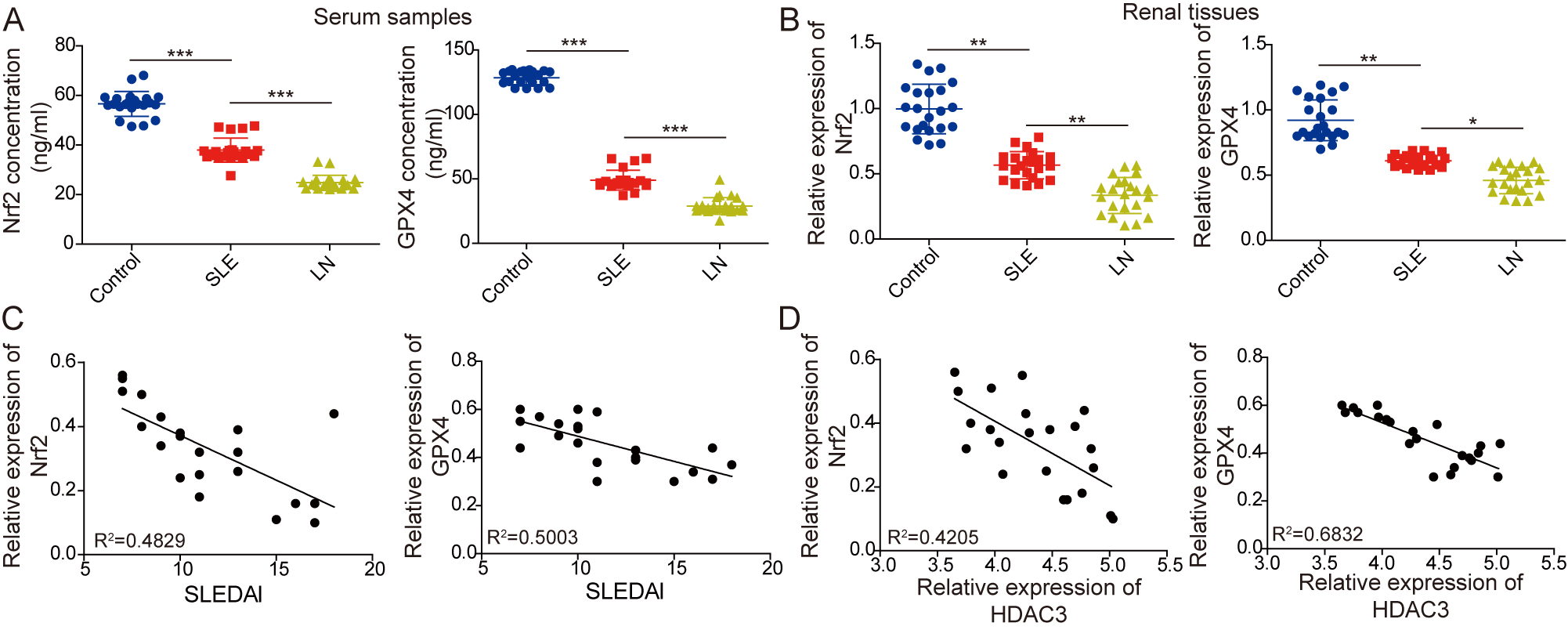


**Figure S1.** **Expression pattern and clinical significance of Nrf2/GPX4 in SLE patients combined with lupus nephritis, with their association with HDAC3 levels.**

(A) Serum Nrf2 and GPX4 levels of SLE, LN, and Control patients were evaluated using ELISA assays. (B) Renal Nrf2 and GPX4 gene expressions of SLE, LN, and Control patients were determined by qRT-PCR analysis. (C) Correlation analysis between renal Nrf2 and GPX4 expressions and SLEDAI scores in the LN group. (D) Correlation analysis between renal Nrf2 and GPX4 expressions and HDAC3 expressions in the LN group. The results obtained are displayed as mean values ± standard deviation (SD). The levels of significance were defined as follows: * indicating *P* < 0.05, ** signifying *P* < 0.01, and *** denoting *P* < 0.001.
