# Supplementary figures and images for: HDAC3 knockdown inhibits ferroptosis via upregulating Nrf2 to alleviate renal interstitial fibrosis in lupus nephritis
Source: Lupus Sci Med. 2025 Oct 22;12(2):e001666. doi: 10.1136/lupus-2025-001666 (PMC12551480; doi:10.1136/lupus-2025-001666)

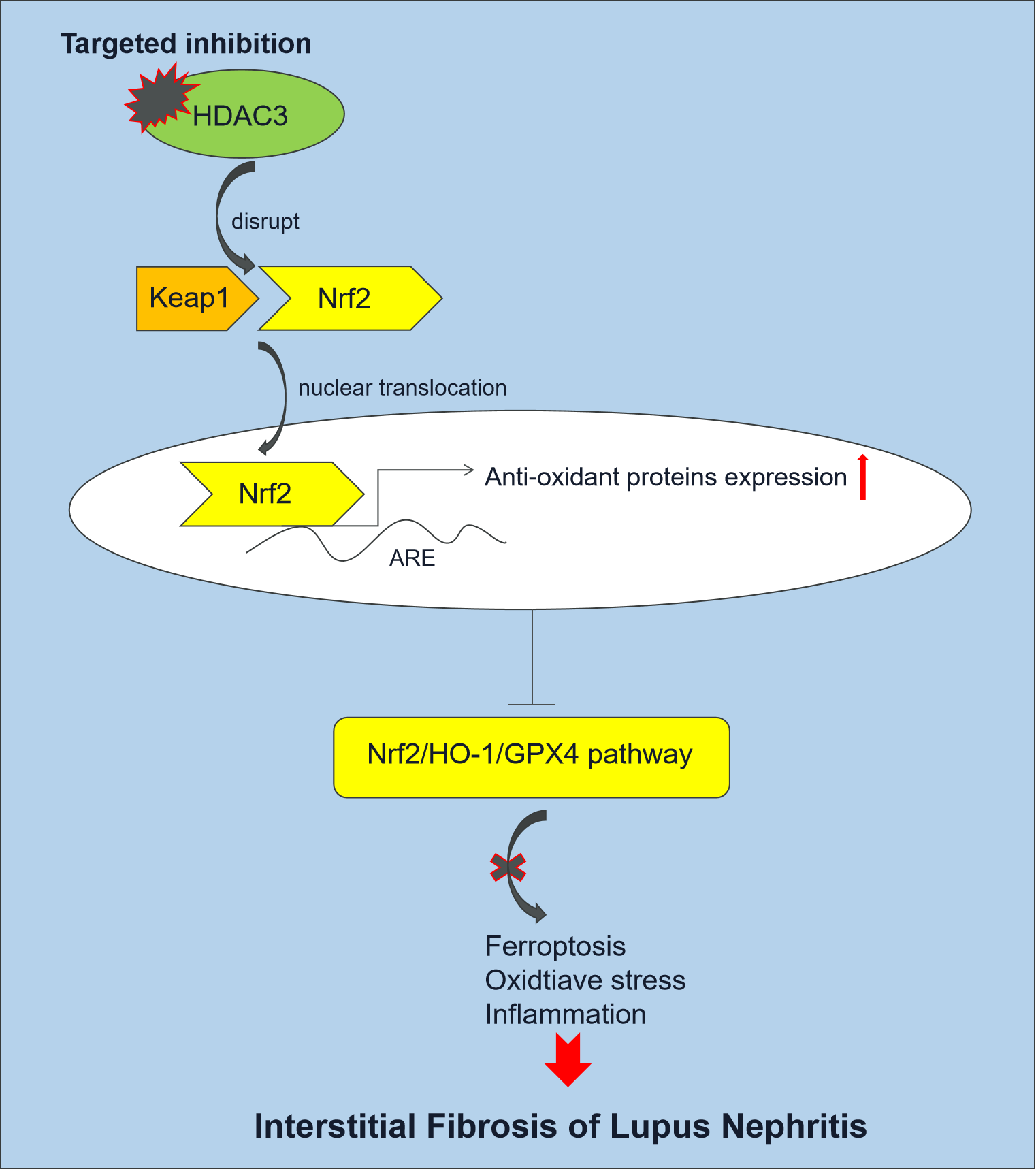

Supplement: online supplemental file 2 [file lupus-12-2-s002.tif]
